# Forced Expression of ZNF143 Restrains Cancer Cell Growth

**DOI:** 10.3390/cancers3043909

**Published:** 2011-10-19

**Authors:** Hiroto Izumi, Yoshihiro Yasuniwa, Masaki Akiyama, Takahiro Yamaguchi, Akihiro Kuma, Noriaki Kitamura, Kimitoshi Kohno

**Affiliations:** Department of Molecular Biology, School of Medicine, University of Occupational and Environmental Health, 1-1 Iseigaoka, Yahatanishi-ku, Kitakyushu 807-8555, Japan; E-Mails: yasuniwa84@yahoo.co.jp (Y.Y.); m-akky@med.uoeh-u.ac.jp (M.A.); t-yama@clnc.uoeh-u.ac.jp (T.Y.); akihiro_k@me.com (A.K.); nk@med.uoeh-u.ac.jp (N.K.); k-kohno@med.uoeh-u.ac.jp (K.K.)

**Keywords:** ZNF143, cell cycle, cell division

## Abstract

We previously reported that the transcription factor Zinc Finger Protein 143 (ZNF143) regulates the expression of genes associated with cell cycle and cell division, and that downregulation of ZNF143 induces cell cycle arrest at G2/M. To assess the function of ZNF143 expression in the cell cycle, we established two cells with forced expression of ZNF143 derived from PC3 prostate cancer cell lines. These cell lines overexpress genes associated with cell cycle and cell division, such as polo-like kinase 1 (*PLK1*), aurora kinase B (*AURKB*) and some minichromosome maintenance complex components (*MCM*). However, the doubling time of cells with forced expression of ZNF143 was approximately twice as long as its control counterpart cell line. Analysis following serum starvation and re-seeding showed that PC3 cells were synchronized at G1 in the cell cycle. Also, ZNF143 expression fluctuated, and was at its lowest level in G2/M. However, PC3 cells with forced expression of ZNF143 synchronized at G2/M, and showed lack of cell cycle-dependent fluctuation of nuclear expression of MCM proteins. Furthermore, G2/M population of both cisplatin-resistant PCDP6 cells over-expressing ZNF143 (derived from PC3 cells) and cells with forced expression of ZNF143 was significantly higher than that of each counterpart, and the doubling time of PCDP6 cells is about 2.5 times longer than that of PC3 cells. These data suggested that fluctuations in ZNF143 expression are required both for gene expression associated with cell cycle and for cell division.

## 1. Introduction

Zinc finger protein 143 (ZNF143) is a transcription factor identified as a human homolog of Staf [1] and is involved in the transcriptional regulation of snRNA and snRNA-type genes by RNA polymerase II or III [2,3]. The 638 amino acid ZNF143 protein contains seven zinc fingers and binds to the YY(A/T)CCC(A/G)N(A/C)AT(G/C)C(A/C)YY sequence in promoter regions [1,4,5]. Functional classification of ZNF143 target genes has revealed that many of these genes are important for cell proliferation [5]. We have previously reported that knockdown of ZNF143 reduces cell proliferation and induces G2M cell cycle arrest. Additionally, we found that ZNF143 knockdown resulted in the downregulation of 152 genes in PC3 human prostate cancer cells. Of these 152 genes, 41 (27%) were associated with cell cycle and DNA replication. In particular, polo-like kinase 1 (*PLK1*), aurora kinase B (*AURKB*) and some minichromosome maintenance complex component (*MCM*) genes were transcriptionally regulated by ZNF143 [6].

We also previously reported that ZNF143 expression is induced by DNA-damaging agents, and is overexpressed in cisplatin-resistant prostate cancer PC3 cell lines [7,8]. However, the proliferation rate of the cisplatin-resistant cell lines is generally slower than that of its parent cell line. To assess the effect of ZNF143 expression on the cell cycle, we established PC3 cells with forced expression of ZNF143, and investigated protein expression associated with cell division, as well as ZNF143 expression according to cell cycle phase. The proliferation of PC3 cells with forced expression of ZNF143 is much slower than that of its wild-type counterpart cell line, and nuclear expression of several MCMs between these cells is different. We hypothesize that the expression cycle of ZNF143 is associated with cell division.

## 2. Results and Discussion

### 2.1. Cell Proliferation of PC3 Cells with Forced Expression of ZNF143

**Figure 1.**
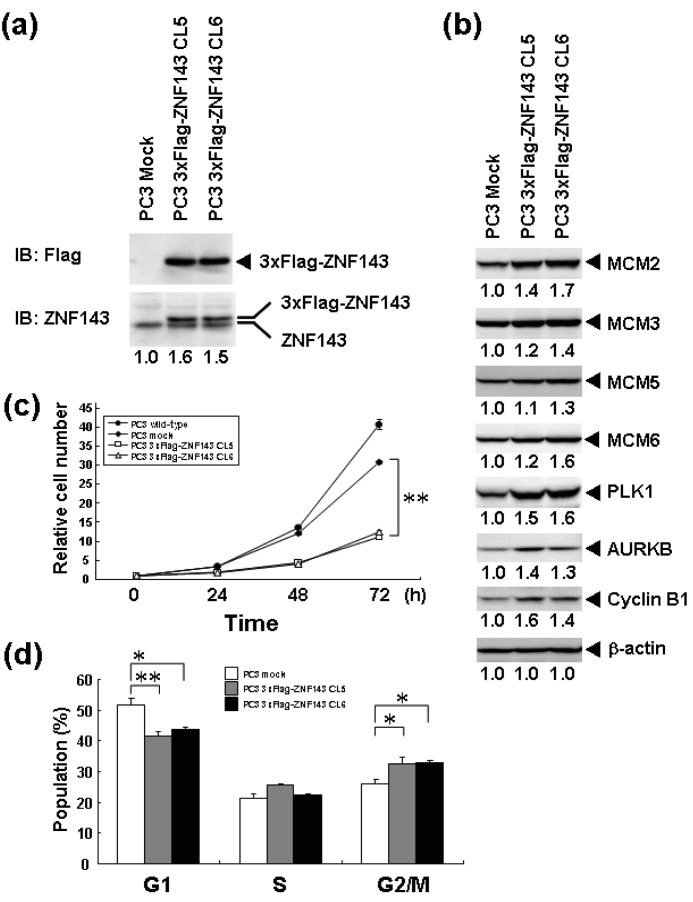
Forced expression of ZNF143 and cell proliferation. (**a**) Whole cell lysates (100 μg) from PC3 cells with forced expression of 3xFlag-ZNF143 and mock transfected cells were used for Western blotting with anti-ZNF143 and anti-Flag antibodies; (**b**) Whole cell lysates (100 μg) of PC3 cells with forced expression of 3xFlag-ZNF143 cells and mock transfected cells were used for western blotting with the indicated antibodies. CL5 and CL6 represent different clones; (**c**) Cells were counted after the indicated times, with time zero being 24 h after seeding. The results were normalized to cell numbers at time zero. The points represent the mean of at least three independent experiments; the bars show the SD. ** *P* < 0.01; (**d**) PC3 mock and cells with forced expression of ZNF143 cells were harvested, stained with propidium iodide and DNA content in single cells was measured by flow cytometry. * *P* < 0.05, ** *P* < 0.01.

We previously reported that knockdown of ZNF143 in PC3 prostate cancer cells reduces the cell proliferation rate, induces G2M cell cycle arrest, and results in downregulation of 41 genes associated with cell cycle and DNA replication [6]. To assess the effect of ZNF143 expression on cell cycle, we established PC3 cells with forced expression of ZNF143 (Figure 1a). Total ZNF143 protein in these cell lines is about 1.5 times higher than that of its counterpart normal cell line. As shown in Figure 1b, cellular expression of PLK1, AURKB, MCM2, MCM3, MCM5, MCM6 and Cyclin B1 is upregulated in PC3 cells with forced expression of ZNF143. These results are consistent with results obtained with ZNF143 siRNA knock down [6]. We predicted that the proliferation rate of these cells might increase according to ZNF143 expression. Unexpectedly, the proliferation rate was much lower than its control counterpart cell line (Figure 1c). The doubling time of these transfectants was then calculated based on these results (Table 1). FACS analysis showed that G1 and G2/M populations of PC3 cells with forced expression of ZNF143 significantly decreased and increased, respectively, compared with PC3 mock cells (Figure 1d). Y box binding protein 1 (YB-1) is also induced by DNA-damaging agents and is overexpressed in cisplatin-resistant cells as well as ZNF143 [9-11]. We reported that YB-1-regulated expression of cell division cycle 6 homolog (CDC6) [11] required for the initiation of DNA replication [12], and downregulation of both YB-1 and CDC6 induces G1 cell cycle arrest [11,13]. However, overexpression of YB-1 also reduces proliferation potency, as observed for ZNF143 [14].

### 2.2. Cell Cycle Profiles of PC3 and Fluctuation in ZNF143 Expression

**Figure 2.**
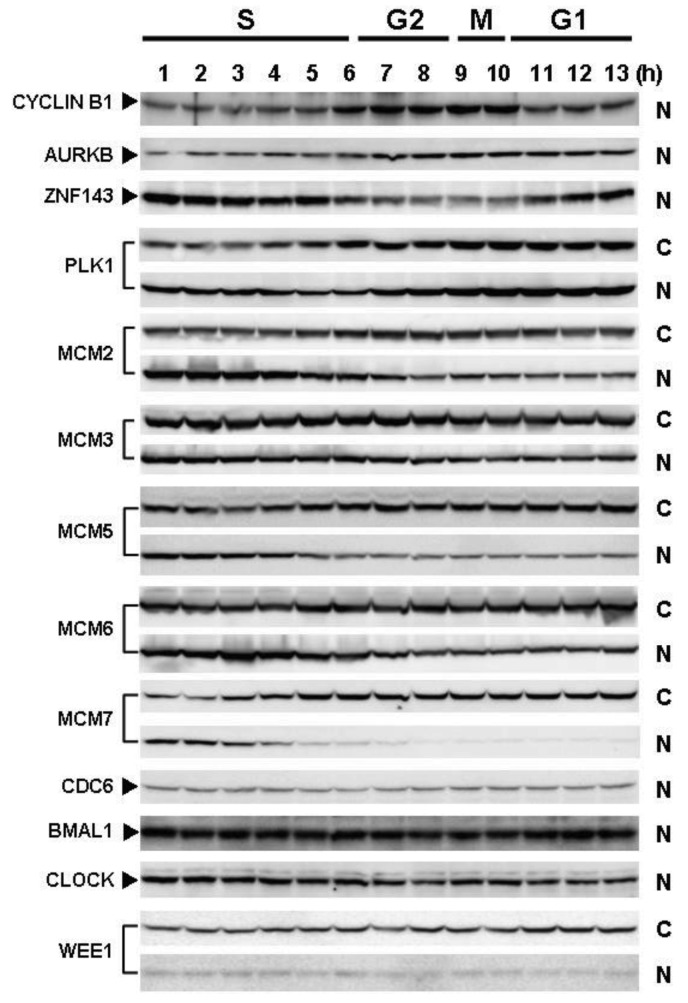
Fluctuations of protein expression associated with the cell cycle in PC3 cells. PC3 cells cultured for 12 h in serum-free medium were re-seeded. At the indicated times after re-seeding, cells were collected and fractionated into nucleoplasm and cytoplasm. Each cell lysate (100 μg) was used for Western blotting with indicated antibodies. N and C indicate nucleoplasm and cytoplasm, respectively.

Cells with forced expression of ZNF143 exhibited the upregulation of PLK1, AURKB and some MCM proteins (Figure 1b), however, proliferation rate was lower than that of its counterpart normal PC3 cells. To clarify this contradiction, we first evaluated the expression of these genes according to cell cycle phase with PC3 cells. As shown in Figure 2, Cyclin B1 expression is higher from 6 h to 10 h and returns to the same expression level as that observed 1 h by 13 h.

This cell cycle phase is consistent with 13.2 h of doubling time (Table 1). It is thought that PC3 cells were synchronized by serum starvation and re-seeding, and 6 h to 10 h represents G2 and M2 phase of the cell cycle. Nuclear expression of AURKB is almost consistent with Cyclin B1 expression, and PLK1 expression in both the cytoplasm and nucleoplasm are increased following induction of Cyclin B1 expression at 6 h. Shindo *et al.* reported that AURKB was expressed during S and G2/M phases [15], and this is almost consistent with our result. A previous study reported that the MCM complex is required for two events of the cell cycle; one is the entry into S phase and the other is cell division [16]. Consistently, we found that MCM protein expression in the nucleoplasm decreased at G2 phase. In particular, MCM4 and MCM7 were hardly observed in nucleoplasm. However, there are no obvious differences in the expression of Clock, Bmal1, Wee1 and CDC6 during the cell cycle phases. These indicate that serum starvation and re-seeding of cells is a straightforward method of synchronizing cells at G1 phase compared with conventional double thymidine block synchronization.

**Figure 3.**
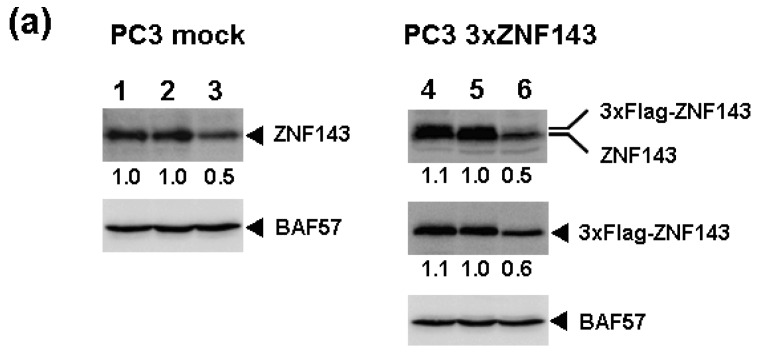

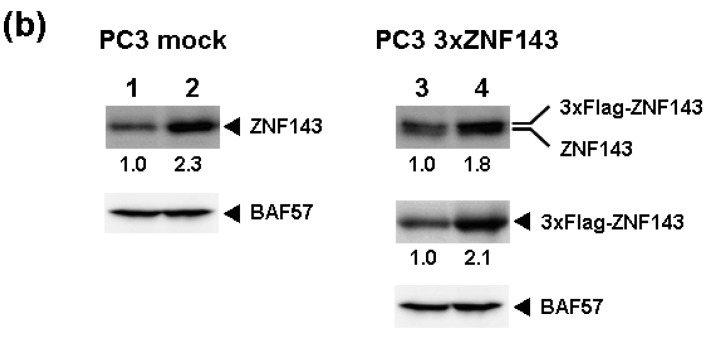
ZNF143 expression under serum condition (**a**) PC3 mock cells and CL5 cells with forced expression of ZNF143 were cultured under serum starvation for 12 h and 24 h, respectively. And re-seeding with medium containing serum was performed. Each nuclear protein (100 μg) was used for western blotting. BAF57 (SWI/SNF related, matrix associated, actin dependent regulator of chromatin, subfamily e, member 1) was used as internal control. Lane 1 and 4; pre-serum starvation, lane 2; serum positive for 12 h, lane 3; serum free for 12 h, lane 5; serum positive for 24 h, lane 6; serum free for 24 h; (**b**) Cells under serum starvation were re-seeded with medium containing serum and collected at indicated time. Each nuclear protein (100 μg) was used for western blotting. BAF57 was used as internal control. Lane 1 and 3; pre-re-seeding, lane 2 and 4; 1 h and 2 h after re-seeding, respectively.

Fluctuations in ZNF143 expression according to cell cycle phase were also observed (Figure 2). First, serum starvation for 12 h decreased ZNF143 expression to 50% (Figure 3a) and re-seeding cells with medium containing serum increased its expression to 2.3-times at 1 h (Figure 3b). After 1 h of re-seeding, ZNF143 gradually decreased until G2/M phase and increased again at the early G1 phase (Figure 2). Interestingly this fluctuation is completely reverse pattern compared with Cyclin B1. CCNB1IP1 (cyclin B1 interacting protein 1, E3 ubiquitin protein ligase) has an activity of E3 ubiquitin protein ligase inducing degradation of Cyclin B1 [17]. ZNF143 may transcriptionally regulate CCNB1IP1 expression to decrease Cyclin B1.

### 2.3. Cell Cycle Profiles of Cells with Forced Expression of ZNF143

**Figure 4.**
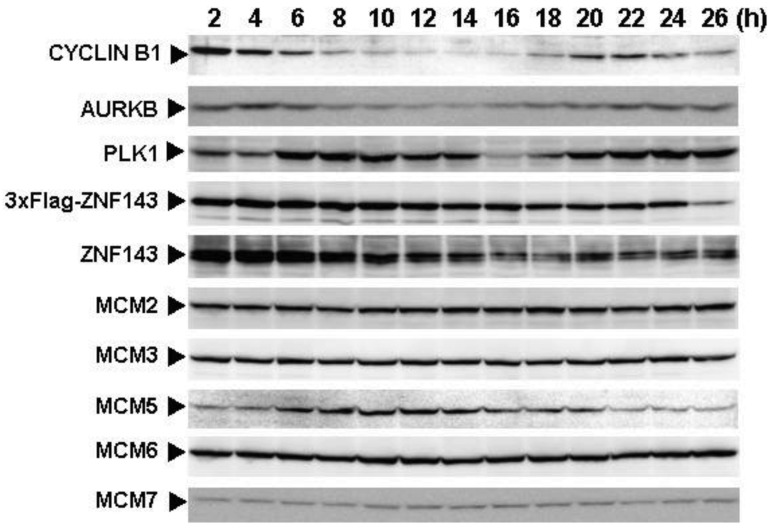
Fluctuations of protein expression associated with the cell cycle in PC3 cells with forced expression of ZNF143. PC3 cells with forced expression of 3xFlag-ZNF143 were cultured for 24 h in serum-free medium and then re-seeded. At the indicated times after re-seeding, cells were collected and fractionated into nucleoplasm and cytoplasm. Each nuclear protein (100 μg) was used for Western blotting with the indicated antibodies.

To assess the effect of ZNF143 on cell cycle, we investigated fluctuations in the expression of Cyclin B1, AURKB, PLK1 and MCM in cells with forced expression of ZNF143. The doubling time of these cells is about 23 h, therefore cells were collected every 2 h until 26 h. Before re-seeding 3xFlag-ZNF143 expression was decreased to 50% by serum starvation (Figure 3a). Exogenous ZNF143 is driven by CMV promoter containing binding motifs of AP1, CREB and NF-kappa B activated by serum stimulation. Actually 3xFlag-ZNF143 expression was increased to 2.1-times at 2 h after re-seeding with medium containing serum (Figure 3b). As shown in Figure 4, 3xFlag-ZNF143 expression continued until 24 h, but endogenous ZNF143 declines gradually with unknown molecular mechanism.

Interestingly, fluctuations in Cyclin B1, AURKB and PLK1 expression were observed twice during 26 h after re-seeding, indicating cells wanted to undergo two rounds of cell cycle. However, we could not find multinuclear in cells with forced expression of ZNF143 by fluorescence microscope with DNA staining (data not shown) and FACS analysis (Figure 1d), namely division is one time during 23 h. On the other hand, Cyclin B1 expression is highest at 2 h after re-seeding indicating that PC3 cells with forced expression of ZNF143 may be synchronized at G2/M phase.

It was reported that knock-down of AURKB [18] or PLK1 [19,20] induce G2/M or mitotic arrest. We previously reported that ZNF143 transcriptionally regulated AURKB and PLK1 and that knock-down of ZNF143 decreased these gene expressions and induced G2/M arrest [6]. G2/M arrest by knock-down of ZNF143 might depend on AURKB and PLK1 expressions. It was reported that knock-down of Aurora-A kinase induces G/2M arrest [21] like AURKB and PLK1, but forced expression of Aurora-A kinase also decreased cell proliferation failing to overcome the restriction point at the G1/S transition due to diminished RB phosphorylation caused by reduced Cyclin D1 expression [22]. In cells with forced expression of ZNF143, AURKB and PLK1 expressions were upregulated (Figure 1b). Constitutive activities of these kinases might deregulate the phosphorylation of several proteins associated with cell cycle and increase G2/M population resulting in deviation from normal division.

**Figure 5.**
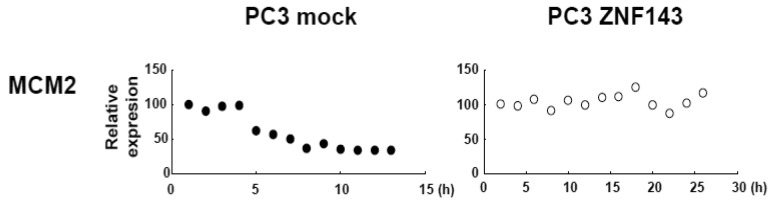

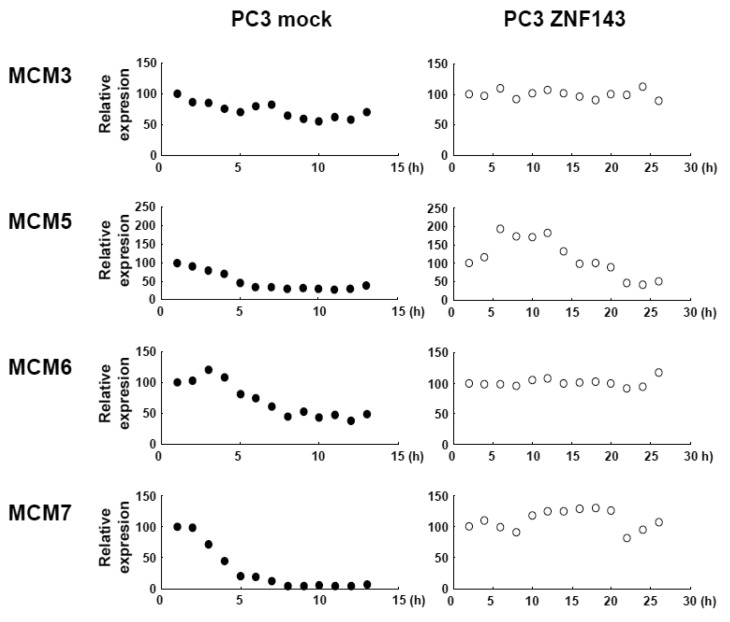
Fluctuations of nuclear expression of MCM proteins. Nuclear MCM2, MCM3, MCM5, MCM6 and MCM7 protein expression levels in Figures 2 and 3 were quantified using Multi Gauge Version 3.0. Closed circles and open circles indicate PC3 mock transfected and PC3 cells with forced expression of ZNF143, respectively.

MCM proteins in the nucleoplasm of PC3 cells decreased or disappeared at G2 phase (Figure 2), those in cells with forced expression of ZNF143 were continuously expressed during cell cycle (Figures 4 and 5). Conversely, MCM5 protein in the nucleoplasm increased. We reported that ZNF143 transcriptionally regulates the expression of *MCM2*, *MCM3*, *MCM5* and *MCM6* [6]. However, the expression profile according to cell cycle phase was not consistent with the expression pattern exhibited by ZNF143 (Figure 2). This result suggests that ZNF143 might be associated with basal transcriptional regulation of these genes. And the cell-cycle dependent fluctuations in the expression of these gene products may be post-translationally regulated. Another possibility is that fluctuations in ZNF143 expression may be associated with localization of MCM proteins. As shown in Figure 2, nuclear expression profile from S to G2/M phase was relatively consistent with the expression pattern exhibited by ZNF143. In any event, these results suggest that Cyclin/CDK pathway and cell division by MCM regulation might be dissociated in cells with forced expression of ZNF143. Further study is necessary to elucidate the molecular mechanism associated with ZNF143 on cell cycle and cell division.

### 2.4. Proliferation of Cisplatin-Resistant Cells

**Figure 6.**
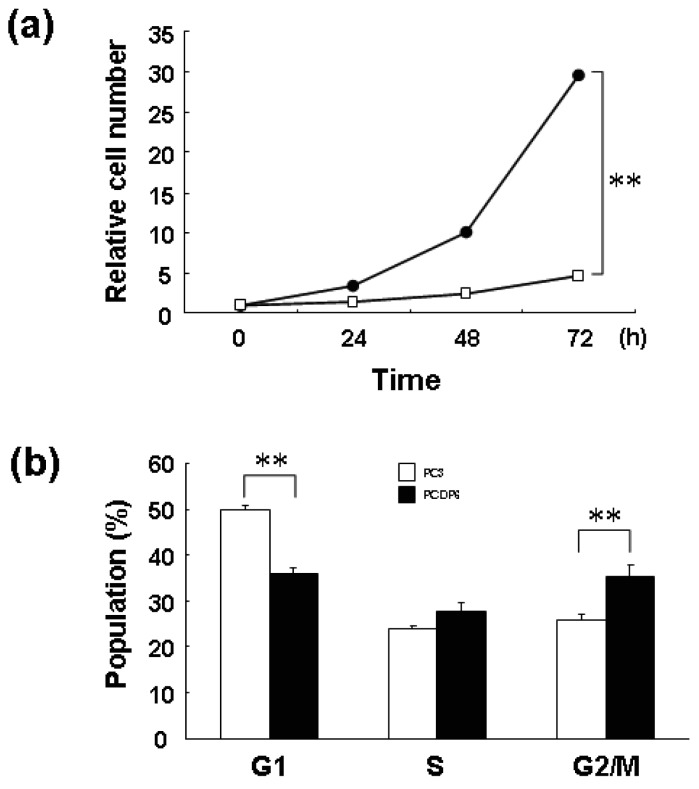
Proliferation rates of PC3 and PCDP6 cell lines. (**a**) Cells were counted at the indicated times, with time zero being 24 h after seeding. Results were normalized to cell numbers at 0 h. The points represent the mean of at least three independent experiments; the bars show the SD. ** *P* < 0.01; (**b**) PC3 and PCDP6 cells were harvested, stained with propidium iodide and DNA content in single cells was measured by flow cytometry. * *P* < 0.05, ** *P* < 0.01.

We previously reported that ZNF143 is overexpressed in cisplatin-resistant PCDP6 cells compared with the parent PC3 cells. Resistance was probably acquired through transcriptional expression of DNA repair-related genes such as APE1 and FEN1 by ZNF143 [8]. First, we investigated the proliferation rate of PC3 and PCDP6 cells. As shown in Figure 6a, the proliferation rate of PCDP6 cells was significantly lower than that of PC3 cells, and the doubling time of these cells was 35.8 h and 13.9 h, respectively.

In general, decrease of cell proliferation is advantageous to resist anticancer agents targeting DNA. Therefore, both expression of repair genes and slow proliferation might be benefit for cisplatin resistance. As shown in Figure 6b, FACS analysis showed that G1 and G2/M populations of PCDP6 cells were significantly lower and higher, respectively. This result of PCDP6 cells is similar to that of cells with forced expression of ZNF143 (Figure 1d). Transition from G2/M to G1 phase might be disturbed in both cells and decrease of ZNF143 might be necessary for cells to pass through the G2/M checkpoint. We reported that cell growth of lung cancer cell lines was significantly correlated with cellular expression of ZNF143 [6]. It is possibility that these lung cancers express ZNF143 with fluctuation but PCDP6 cells continually overexpressed ZNF143. In either case, both downregulation of ZNF143 and forced expression of ZNF143 might induce G2/M arrest or increase G2/M population to repress the cell proliferation. These results suggest that overexpression of ZNF143 with fluctuation is different from forced expression of ZNF143 on cell cycle. Further study is necessary to elucidate the molecular mechanism associated with ZNF143 on drug resistance.

## 3. Experimental Section

### 3.1. Cell Culture and Antibodies

Human prostate cancer cells, PC3 [8], and cisplatin-resistant PC3 cells, PCDP6 cells, were cultured in Minimum Essential Medium containing 10% fetal bovine serum. Cell lines were maintained in a 5% CO2 atmosphere at 37 °C. Antibodies against Flag (M2) and β-actin (A5441) were purchased from Sigma Aldrich (St. Louis, MO, USA). Antibodies against MCM2 (N-19), MCM3 (N-19), MCM5 (H-300), MCM6 (C-20), MCM7 (1412), Cyclin B1 (H-433) and Clock (M-20) were purchased from Santa Cruz Biotechnology (Santa Cruz, CA, USA). Antibodies against PLK1 (37-7000), CDC6 (05-550), aurora B kinase (AURKB) (1788-1) and Wee1 (#4936) were purchased from Invitrogen (Carlsbad, CA, USA), Upstate Biotech (Lake Placid, NY, USA), Epitomics (Burlingame, CA, USA) and Cell Signaling Technology (Beverly, MA, USA), respectively. Production of polyclonal antibody against ZNF143 and BAF57 were described previously [6]. Polyclonal antibody against Bmal1 was raised by multiple immunizations of a New Zealand white rabbit with synthetic peptides. The synthetic peptide sequence was LGGPVDFSDLPWPL.

### 3.2. Establishment of PC3 Cells Stably Expressing ZNF143

Establishment and cloning of PC3 cells with forced expression of ZNF143 by transfection with 3xFlag-ZNF143 expression plasmid was described previously [8]. PC3 mock cells were transfected with empty expression plasmid and selected by hygromycin without cloning.

### 3.3. Cell Proliferation Assays and Doubling Time

Cell proliferation assay was described previously [6]. Briefly, PC3, PCDP6 and PC3 cells with forced expression of ZNF143 were seeded into 12-well plates at a density of 1 × 10^4^ cells per well. Cells were harvested by trypsinization and counted every 24 h with a Coulter-type cell size analyzer (CDA-500; Sysmex Corp., Kobe, Japan). The first measurement time was set as time zero. Proliferation curve graph was converted into log function and doubling-time was calculated.

### 3.4. Western Blotting and Cell Cycle Expression Profiling

The preparation of cytoplasmic and nuclear proteins was described previously [6,8]. The indicated amounts of whole cell lysate, cytoplasmic protein (cytoplasm) and nuclear protein (nucleoplasm) were subjected to Western blotting and detection was performed using enhanced chemiluminescence (Amersham, Piscataway, NJ, USA). Protein levels were quantified using Multi Gauge Version 3.0 (Fujifilm, Tokyo, Japan). For synchronization of the cell cycle, the serum starvation and re-seeding method was employed. PC3 cells and PC3 cells with forced expression of ZNF143 were washed twice with PBS and cultured in serum-free medium for 12 h and 24 h, respectively. Cells were disaggregated with trypsin and re-seeded in culture dishes with medium containing 10% fetal bovine serum. At the indicated times after re-seeding, cells were collected and proteins were prepared.

### 3.5. Flow Cytometry Analysis

Flow cytometry was described previously [23]. Briefly, siRNA-transfected PC3 cells (1 × 10^6^) were seeded into 90-mm plates. After 72 h, the cells were analyzed using an EpicsXL-MCL flow cytometer (Beckman-Coulter, Miami, FL, USA).

### 3.6. Statistical Analysis

Student t test was used for statistical analysis of the variables between the two groups. All error bars indicate standard deviation.

## 4. Conclusions

We found fluctuations in ZNF143 expression with prostate cancer PC3 cells. Either down-regulation of ZNF143 or forced overexpression of ZNF143 decreased cell proliferation. Cell growth is sometimes associated with the efficacy of anti-cancer agents targeting DNA, because disturbance of DNA replication decreased when cell proliferation is slow. We established several resistant cells against anticancer agents and growth rates of these cells are almost all low. However, molecular mechanism is unknown. Cisplatin resistant prostate cancer cells grow slowly with overexpression of ZNF143 and increase of G2/M population. These results similar to cells with forced expression of ZNF143. Either forced increase or forced decrease of cell cycle-related genes might induce the disturbance of cell division. We believe that ZNF143 is a promising molecular target to overcome cancers. Deregulation of the cell cycle-dependent fluctuation in ZNF143 expression also might prevent the cancer proliferation even if cancer cells maintain strong expression of ZNF143. Further analysis of interplay between ZNF143 and other cell cycle regulators is required to understand the essential role of ZNF143 in cell cycle and drug sensitivity.

## Figures and Tables

**Table 1.** Doubling time of wild-type PC3 cells, PC3 cells with forced expression of 3xFlag-ZNF143 and mock transfected cells.

	**Doubling time (h)**
PC3 wild-type	13.2
PC3 mock	13.3
3xFlag-ZNF143 CL5	22.9
3xFlag-ZNF143 CL6	23.8
